# Does cognitive frailty predict delayed neurocognitive recovery after noncardiac surgery in frail elderly individuals? Probably not

**DOI:** 10.3389/fnagi.2022.995781

**Published:** 2022-11-15

**Authors:** Jingya Zhang, Diksha Basnet, Xue Du, Junjun Yang, Jiehui Liu, Fan Wu, Xiaoqing Zhang, Jianhui Liu

**Affiliations:** Department of Anesthesiology, School of Medicine, Tongji Hospital, Tongji University, Shanghai, China

**Keywords:** cognitive frailty, delayed neurocognitive recovery, nomogram, predictive model, frail elderly

## Abstract

**Introduction:**

Delayed neurocognitive recovery (DNR) is a common post-surgical complication among the elderly. Cognitive frailty (CF) is also an age-related medical syndrome. However, little is known about the association between CF and DNR. Therefore, this study aimed to study whether CF is associated with DNR in elderly patients undergoing elective noncardiac surgery, as well as to explore the potential risk factors for DNR in frail elderly individuals and construct a prediction model.

**Methods:**

This prospective cohort study administered a battery of cognitive and frailty screening instruments for 146 individuals (≥65 years old) scheduled for elective noncardiac surgery. Screening for CF was performed at least one day before surgery, and tests for the presence of DNR were performed seven days after surgery. The association between CF and DNR was investigated. Moreover, the study subjects were randomly divided into a modeling group (70%) and a validation group (30%). Univariate and multivariate logistic regression was performed to analyze the modeling group data and identify the independent risk factors for DNR. The R software was used to construct DNR's nomogram model, verifying the model.

**Results:**

In total, 138 individuals were eligible. Thirty-three cases were diagnosed with DNR (23.9%). No significant difference in the number of patients with CF was observed between the DNR and non-DNR groups (*P* > 0.05). Multivariate analysis after adjusting relevant risk factors showed that only the judgment of line orientation (JLOT) test score significantly affected the incidence of DNR. After internal validation of the constructed DNR prediction model, the area under the curve (AUC) of the forecast probability for the modeling population (*n* = 97) for DNR was 0.801, and the AUC for the validation set (*n* = 41) was 0.797. The calibration curves of both the modeling and validation groups indicate that the prediction model has good stability.

**Conclusion:**

Cognitive frailty is not an independent risk factor in predicting DNR after noncardiac surgery in frail elderly individuals. The preoperative JLOT score is an independent risk factor for DNR in frail elderly individuals. The prediction model has a good degree of discrimination and calibration, which means that it can individually predict the risk probability of DNR in frail elderly individuals.

## Introduction

In China, with the increased life expectancy, approximately 1/3 of elective surgical patients are elderly (≥65 years old) (Culley et al., 2017). Advances in surgical and anesthesia techniques, coupled with better preoperative risk assessment, have resulted in safer operations and lower rates of some serious complications (e.g., hemorrhage, infections) (Kim et al., 2015); however, much less is known about effectively protecting the aging brain from perioperative stress.

Delayed neurocognitive recovery (DNR) is a prevalent post-surgical complication in the elderly, with an incidence of 10–65% in geriatric surgery patients (Androsova et al., 2015; Boone et al., 2020). It is characterized by a temporary disruption in the patient's memory, personality, sleep, or executive function, which can manifest weeks or months following surgery. It may persist for months or even more (Needham et al., 2017). Age, education level, and preoperative cognitive status have been identified as risk factors (Kotekar et al., 2018). It has been proven to be linked with several unfavorable outcomes, including extended hospital stays, increased unanticipated complications, and mortality, which can lead to higher healthcare expenses and degrade patients' quality of life, placing a heavy burden on patients and their families (Monk et al., 2008). However, at present, much less is known about etiopathogenesis and therapies for DNR. There are no effective measures to protect the aging brain from DNR. Therefore, identifying and avoiding its risk factors might be an efficient strategy for therapy (Li et al., 2021).

Moreover, with the advancement of age, the risk of the incidence of frailty increases. The prevalence of frailty in the elderly ranges from 12 to 59% (Collard et al., 2012; O'Caoimh et al., 2021). “Fragile and vulnerable settings” have been listed as one of the top ten global health threats by the WHO since 2019 (Uslu and Canbolat, 2021). Frailty is an age-related medical syndrome that usually leads to a loss of reserve capacity to respond to stressors, deterioration of bodily functions and systems, and dependence on other people for daily activities, eventually leading to death and morbidity (Zaslavsky et al., 2013). Cognitive impairment is also considered a component of frailty. Based on a large body of evidence supporting a significant association between frailty and cognition (Dartigues and Amieva, 2014), the concept of cognitive frailty used in this study was first defined by the International Consensus Group at the International Academy on Nutrition and Aging (I.A.N.A) and the International Association of Gerontology and Geriatrics (I.A.G.G). Cognitive frailty (CF) is a geriatric syndrome characterized by the concurrence of cognitive impairment and physical frailty without dementia (Bu et al., 2021). The prevalence of cognitive frailty in clinical settings is estimated to be 10.7 to 39.7% (Sugimoto et al., 2018). There was more increment in white matter hyperintensity in patients with cognitive frailty than in those without (Sugimoto et al., 2019). Some evidence indicates that WMH causes cognitive decline and increases the risk of dementia (Prins and Scheltens, 2015). Nevertheless, whether cognitive frailty is associated with DNR has not been demonstrated.

To investigate the possible association of the occurrence of DNR with preoperative cognitive frailty and to explore the potential risk factors for DNR in frail elderly patients who undergo elective noncardiac surgery, we implemented a prospective cohort study of senior individuals who were screened for frailty using the FRAIL scale, which is a brief questionnaire that classifies individuals as frail, pre-frail, and robust; we also used the Montreal Cognitive Assessment (MoCA), which is used to identify mild cognitive impairment (MCI) (Aprahamian et al., 2017). This study aims to investigate the association between cognitive frailty and DNR and establish a prediction model for DNR. We hypothesized that cognitive frailty would correlate with cognitive decline in the early stage.

## Materials and methods

### Study design and participants

**Figure 1:**
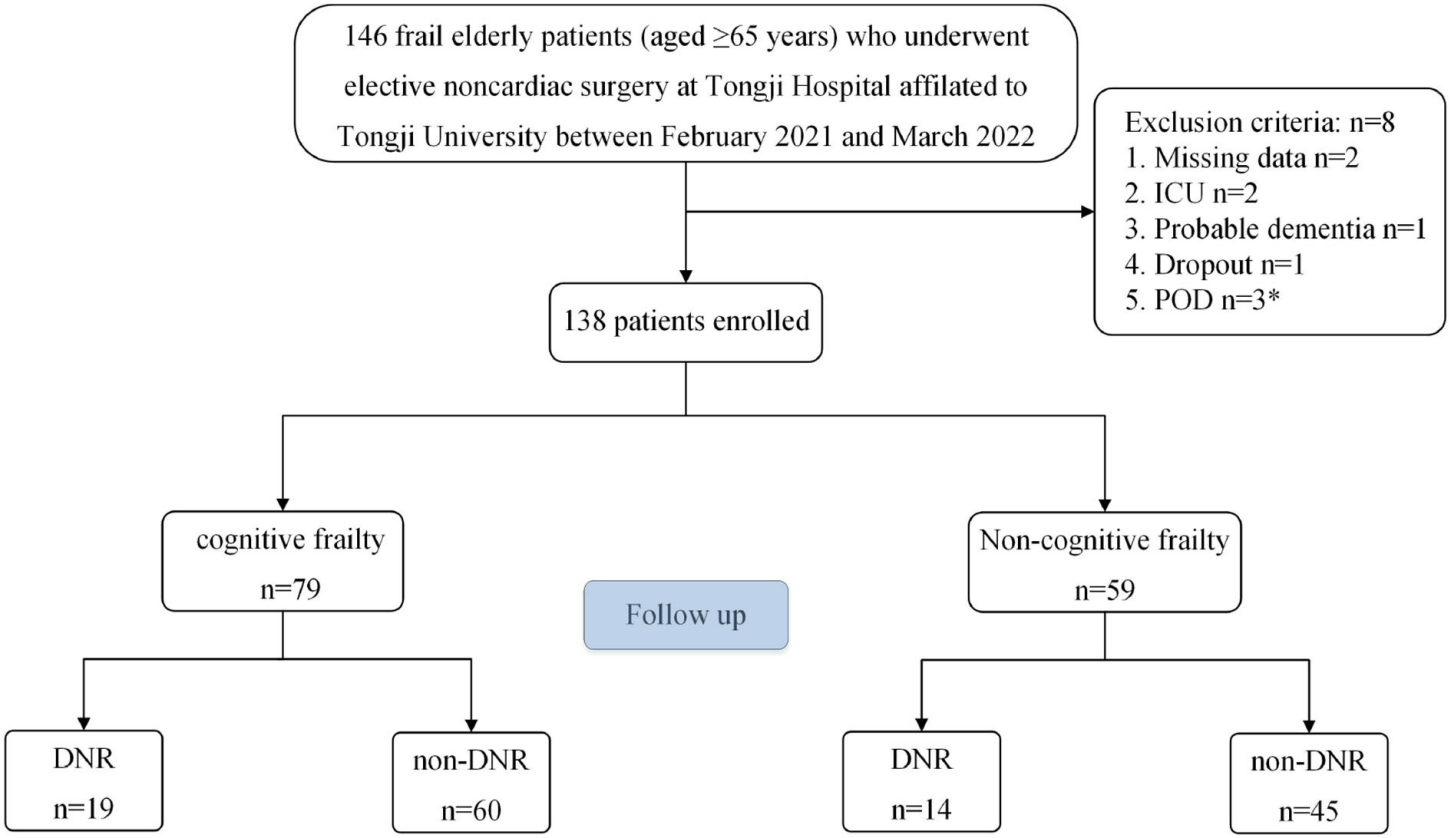
Flow diagram of patient selection process. *Including an ICU patient.

We collected 146 frail elderly patients (aged ≥ 65 years) undergoing elective noncardiac surgery at Tongji Hospital, affiliated with Tongji University, between February 2021 and March 2022 (Figure 1). All eligible patients were asked to provide informed consent. The following inclusion criteria were used: (1) aged ≥65 years; (2) each patient was diagnosed as being frail, FRAIL scale scores ≥3; (Aprahamian et al., 2017); (3) Preoperative Mini-Mental State Examination (MMSE) scores ≥ 20 points; (4) patients were scheduled for elective noncardiac surgery under general anesthesia; and (5) ASA classification I-III. The exclusion criteria were as follows: (1) history of cerebrovascular disease; (2) history of sedatives and antidepressants; (3) history of severe neurological or psychiatric disease; (4) serious audiovisual impairments that impacted assessments; (5) unwillingness to abide by a protocol or withdrawal; (6) Patients admitted to intensive care unit (ICU) after surgery; (7) and patients who developed postoperative delirium (POD). Finally, 138 frail elderly patients were enrolled in this study. All patients were divided into modeling and validation populations using a computer-generated simple randomization list with a 7:3 allocation.

Tongji University's Institutional Review Board approved this study (IRB2021-LCYJ-014), and all subjects participating in the trial obtained written informed consent. The trial was registered before patient enrollment at http://www.chictr.org.cn (ChiCTR2100043475, Principal investigator: Jianhui Liu, registration date: February 19, 2021). All methods were carried out in conformity with the relevant guidelines and regulations. The study subjects gathered data after obtaining written informed consent to participate in the investigation. For the subjects unable to give written informed consent themselves, it was obtained from the legal representatives or guardians of minor subjects.

### Data collection

The clinical characteristics, which were used as perioperative variables, consisted of the information obtained from all the patient's medical chart records or interviews: (1) demographics and clinical baseline data, including gender, age, body mass index (BMI), education, MMSE score, MoCA score, comorbidities (hypertension, diabetes, cardiovascular disease, chronic pulmonary diseases, and others); (2) main clinical parameters, including the type of surgeries (spinal, urinary and gastrointestinal), operation time, anesthesia time, VAS scores (1st, 12th, 24th, 48th h after surgery), Resmay sedation scores (1st, 12th, 24th, 48th h after surgery); (3) Results of 11 neuropsychological tests (NPTs), including one day before surgery and 7 days following the surgery.

### Frailty assessment

While recruiting the patients, trained research coordinators evaluated physical frailty using the FRAIL scale. The FRAIL scale was validated in diverse groups of patients. A screening tool was easily carried out by various medical professionals or caregivers (Abellan Van Kan et al., 2008a,b). Compared with other frailty scores, the FRAIL scale has higher sensitivity and has been associated with mortality and complications. In this study, the FRAIL scale was composed of five components: fatigue, resistance, ambulation, illness, and weight loss. The scale defined “fatigue” as the immanent feeling of tiredness “most or all” in the last 4 weeks. If the patient had difficulty or could not climb a flight of stairs, the “resistance” score was positive. If the patient had difficulty walking a block, the “ambulation” score is positive. The presence score for “illnesses” was positive if the patient had five or more comorbidities, including hypertension, diabetes, congestive heart failure, heart attack, angina, chronic pulmonary disease, asthma, arthritis, stroke, cancer, and nephrological disease. If the patient had an unintentional weight loss of more than 5% in the past year, the “loss of weight” score was positive. A score of 1 was set for each positive domain. Participants were classified as frail if they scored three or above, prefrail if they scored 1–2, and a score of 0 was considered robust (Ritt et al., 2017; Aguayo et al., 2018).

### Cognitive function assessment

#### Cognitive frailty measurement

Cognitive frailty was defined as the combination of physical frailty and MCI, which is manifested by evident changes in at least one cognitive domain while maintaining the independence of functional abilities, and excluding concurrent Alzheimer's disease or other forms of dementia (Kelaiditi et al., 2013). In our study, we screened MCI patients with the MoCA scale. Studies have demonstrated that MoCA has advantages in detecting MCI in the elderly (Pinto et al., 2019). The MoCA is an objective neuropsychologic test that assesses a range of cognitive domains supporting independent functioning, including visuospatial/executive, naming, memory, attention, language, abstraction, delayed recall, and orientation to time and place (Freitas et al., 2012; Horton et al., 2015). It is significantly better at detecting MCI among aged people (Pinto et al., 2019). Compared with MMSE, a general cognitive tool, MOCA has higher sensitivity (90 vs. 18%) and considerable specificity (87 vs. 100%). In contrast, participants were considered to have MCI if their MoCA scores were < 26 points (Nasreddine et al., 2005).

#### Definition of DNR

function assessment was performed at least one day before the surgery (baseline) and on postoperative day 7. Patients were first screened with MMSE and MoCA to eliminate those with dementia and identify those with MCI, respectively. Furthermore, eight tests with 11 subscales were administered, including the Hopkins Verbal Learning Test (HVLT immediate and delayed), Brief Visuospatial Memory Test-revised (BVMT immediate and delayed), Digits Symbol Substitution Test (DSST), Trail Making Test (TMT), Semantic Fluency Test (SFT), Forward and Backward Digit Span Test (DST forward and backward), Stroop Color Word Test (STROOP), and Judgment Of Line Orientation Test (JLOT), mainly focusing on memory, attention force, and executive function, as was the case in our previous study (Du et al., 2021). These measures were not only highly sensitive to the many forms of cognitive impairment but also free of cultural bias. All the tests were conducted in a quiet environment by professionally trained personnel. DNR was defined as a Z score (at least two Z-scores in individual tests, or the composite Z-score of all variables) at ≥ 1.96, as described in the International Study of Post-Operative Cognitive Dysfunction (ISPOCD) before (Moller et al., 1998; Du et al., 2021).

#### Postoperative delirium assessment

Trained members of the study team were assessed for delirium twice daily in the ward (08:00 and 20:00) using the Confusion Assessment Method (CAM) while hospitalized or until day 7. Because POD is a fluctuating state that frequently occurs in the ward and at night, we used chart review to detect any undiagnosed POD episodes (Inouye et al., 2005). Trained researchers reviewed the medical charts. We reviewed the individuals' medical charts to see if the nurse or doctor had any reports of POD (e.g., aggressive or inappropriate behavior, confusion, reports of hallucinations, etc.).

### Anesthesia and postoperative analgesia management

We mainly chose major spine, urology, and gastrointestinal surgery operations. No premeditations were administered before surgery. All inpatients received general anesthesia after obtaining informed consent. Patients were intubated under general anesthesia, using etomidate, sufentanil, and cis-atracurium for anesthesia induction; they were then intubated under a visual laryngoscope. For intraoperative anesthetic maintenance, we chose volatile anesthetic sevoflurane and intravenous anesthetic (propofol and remifentanil).

Meanwhile, cis-atracurium was intermittently injected as needed until the end of surgery. In addition to a routine electrocardiogram, arterial blood pressure, end-expiration carbon dioxide, and blood oxygen saturation, anesthesia depth was monitored during the operation to maintain a bispectral index (BIS) between 40 and 60. Intraoperative blood pressure was controlled, and vasoactive agents were used as needed at the discretion of the attending anesthesiologists. A patient-controlled analgesia device (PCA) was used for postoperative pain management. Sufentanil was the main analgesic agent, and the commonly used dose was 100 μg (diluted with 100 mL of normal saline). Postoperative pain was assessed on a 0–10 visual analog scale (VAS; VAS: 0 = no pain and 10 = worst pain) to keep the VAS below 3 points.

### Statistical analysis

Statistical analyses were performed using SPSS statistics version 26.0 (IBM, Inc., Armonk, NY, USA), and the R programming language was used to perform statistical analyses. Several methods were used to analyze all the data.

We first assessed the normality and outliers of the data using the Shapiro-Wilk Normality Test. Patients were divided into two groups based on the occurrence of DNR: the DNR group and the non-DNR group. We applied appropriate statistical tests to compare differences between groups. When comparing the baseline characteristics, we used either the independent sample *t*-test for normally distributed continuous variables (data expressed as mean ± SD) or the Mann–Whitney U-test for non-normally distributed variables [data expressed as median (25, 75 percentile)]. The categorical variables were used in the chi-square test [data reported as counts (%)]. Statistical significance was set as *P* < 0.05 for all analyses.

A univariate and multivariate binary logistic regression was conducted to analyze risk factors and DNR in the modeling population, and the nomogram prediction model was fitted. Relevant factors of *P*-value ≤ 0.15 in univariate analysis and previously suggested risk factors like age, education, and cognitive impairment (Kotekar et al., 2018) were also included in the multivariate binary logistic regression model. The enter method was used. The odds ratio (OR) and its 95% confidence interval (CI) were used to show relative risk. The internal validation method was used mainly to verify the prediction model. We adopted the receiver operating characteristic curve (ROC) analysis to evaluate the resolution of the prediction model. When the area under the curve (AUC) value was ≥ 0.7, the model was considered to have good discrimination ability. The calibration degree of the prediction model was evaluated by the Hosmer-Lemeshow test and calibration curve (Paul et al., 2013), in which we used 100-repetition bootstrapping to draw calibration curves with the test level α = 0.05. The cut-off point's value was calculated as per the maximum value of Youden's index.

## Results

### Demographics and clinical baseline data

The demographic and clinical features of individuals in the DNR and non-DNR groups are outlined in Table 1. The final analysis included 138 frail elderly patients (≥65 years old). Among them, 33 patients were diagnosed with DNR, with an incidence rate of 23.9% (33/138). There was no significant difference in the number of patients with cognitive impairment between the DNR group (57.6%) and the non-DNR group (57.1%; *P* > 0.05).

**Table 1:** Demographics and clinical data associated with DNR in modeled patients.

**Item**	**DNR**	**non-DNR**	***P*-value**
	**(*n* = 33)**	**(*n* = 105)**	
Male (*n*, %)	23(69.7%)	65(61.9%)	0.417
Age (y), median [Q25, Q75]	69[65, 74]	70[66, 72]	0.872
Education (y), median [Q25, Q75]	9[8, 12]	9[7, 12]	0.476
BMI (kg/m^2^), mean ± SD	24.6 ± 3.2	24.4 ± 3.6	0.720
Surgery time (min), median [Q25, Q75]	165[147, 207]	170[135, 217]	0.648
Anesthesia time (min), median [Q25, Q75]	210[183, 240]	205[170, 268]	0.875
BIS, mean ± SD	50.9 ± 5.4	50.3 ± 5.8	0.583
Cognitive frailty	19(57.6%)	60(57.1%)	0.965
Preoperative MMSE scores, median [Q25, Q75]	28[26, 29]	28[26, 29]	0.861
Preoperative MoCA scores, mean ± SD	24.7 ± 3.4	24.0 ± 3.8	0.301
Cognitive frailty (*n*, %)	12(54.5%)	41(54.7%)	0.992
**Postoperative VAS scores, median [Q25, Q75]**			
1h	2[1, 3]	2[2, 3]	0.010*
12h	1[1, 2]	1[1, 2]	0.337
24h	0[0, 1]	1[0, 1]	0.077
48h	0[0, 0]	0[0, 0]	0.362
**Postoperative Resmay scores, median [Q25, Q75]**			
1h	1[1,2]	1[1,2]	0.457
12h	3[3,3]	3[3,3]	0.182
**Medical history (** ***n*** **, %)**			
Hypertension	18(54.5%)	55(52.4%)	0.828
Diabetes	6(21.2%)	19(18.1%)	0.690
Cardiovascular Disease	3(9.1%)	22(22.9%)	0.843
Chronic Pulmonary Diseases	1(3.0%)	5(0.1%)	0.724
**Surgery category (** ***n*** **, %)**			
Spine	18(54.5%)	54(52.4%)	0.755
Urinary	10(30.3%)	23(21.9%)	0.324
Gastrointestinal	5(15.2%)	28(26.7%)	0.176

BMI, body mass index; MMSE, mini-mental state examination; MoCA, montreal cognitive assessment; and VAS, visual analog scale.

### Risk factors associated with DNR

Table 2 presents the results of univariate and multivariate binary logistic regression analyses for the incidence of DNR in the model population (*n* = 97). The postoperative VAS scores of 1h (OR 0.501, 95%CI 0.267–0.939), the baseline DSST scores (OR 1.057, 95% CI 1.009–1.106), and JLOT scores (OR 1.227, 95% CI 1.016–1.483) were identified as predictors of DNR after surgery. In the multivariate model, the preoperative JLOT score (OR 1.311, 95% CI 1.051–1.636) still significantly influenced DNR even after adjustment for age, education, and cognitive frailty.

**Table 2:** Univariate and multivariate logistic regression analysis of incidence of DNR after elective noncardiac surgery in frail elderly modeling population.

	**Univariate regression analysis**			**Multivariate regression analysis** ^**a**^		
	**OR**	**95%CI**	***p-*value**	**OR**	**95%CI**	***p*-value**
Sex (male)	1.778	0.625–5.059	0.281			
Age (y)	1.05	0.942–1.169	0.378	1.102	0.970–1.253	0.137
Education (y)	1.056	0.898–1.241	0.510	1.048	0.831–1.321	0.692
BMI (kg/m^2^)	1.016	0.896–1.152	0.804			
Surgery time (min)	1.002	0.996–1.008	0.562			
Anesthesia time (min)	1	0.994–1.006	0.976			
BIS	0.994	0.913–1.083	0.897			
Preoperative MMSE scores	1.056	0.826–1.351	0.662			
Preoperative MoCA scores	1.004	0.911–1.195	0.536			
Cognitive frailty (n)	1.005	0.387–2.610	0.992	0.296	0.074–1.191	0.087
**Postoperative VAS scores**
1h	0.501	0.267–0.939	0.031^*^	0.559	0.241–1.298	0.176
12h	0.807	0.452–1.440	0.467			
24h	0.16	0.210–1.294	0.16			
48h	0.889	0.262–3.018	0.85			
**Postoperative Resmay scores**
1h	1.305	0.493–3.455	0.592			
12h	1.856	0.490–7.035	0.363			
**Medical history (n)**
Hypertension	0.943	0.363–2.450	0.904			
Diabetes	0.559	0.184–1.701	0.306			
Cardiovascular Disease	2.784	0.589–13.172	0.197			
Chronic Pulmonary Diseases	1.5	0.166–13.561	0.718			
Surgery category						
spine	1.108	0.427–2.875	0.834			
urinary	2.107	0.753–5.899	0.156			
Gastrointestinal	0.275	0.059–1.284	0.101	1.842	0.306–11.087	0.505
**Baseline cognitive tests scores**
HVLT (immediate)	1.075	0.977–1.181	0.137	0.95	0.794–1.137	0.575
HVLT (delayed)	1.177	0.992–1.397	0.062	1.153	0.836–1.591	0.386
BVMT (immediate)	0.988	0.926–1.055	0.724			
BVMT (delayed)	1.047	0.916–1.197	0.503			
DSST	1.057	1.009–1.106	0.019^*^	1.049	0.986–1.115	0.129
TMT	0.995	0.979–1.013	0.605			
DST (Forward)	0.927	0.783–1.096	0.374			
DST (Backward)	0.966	0.771–1.210	0.763			
SFT	1.066	0.959–1.185	0.238			
STROOP	1.024	0.990–1.059	0.164			
JLOT	1.227	1.016–1.483	0.034^*^	1.315	1.054–1.640	0.015^*^

BMI, body mass index; BVMT, brief visuospatial memory test-revised; DDST, digits symbol substitution test; DST, digit span test; HVLT, Hopkins verbal learning test; JLOT, judgment of line orientation test; MMSE, Mini-mental state examination; MoCA, montreal cognitive assessment; SFT, semantic fluency test; STROOP, stroop color word test; TMT, trail making test; and VAS, visual analog scale.

a Adjusted for age, education, and cognitive frailty.

* *P* < 0.05.

### Predictive performance analysis of the DNR prediction model

**Figure 2:**
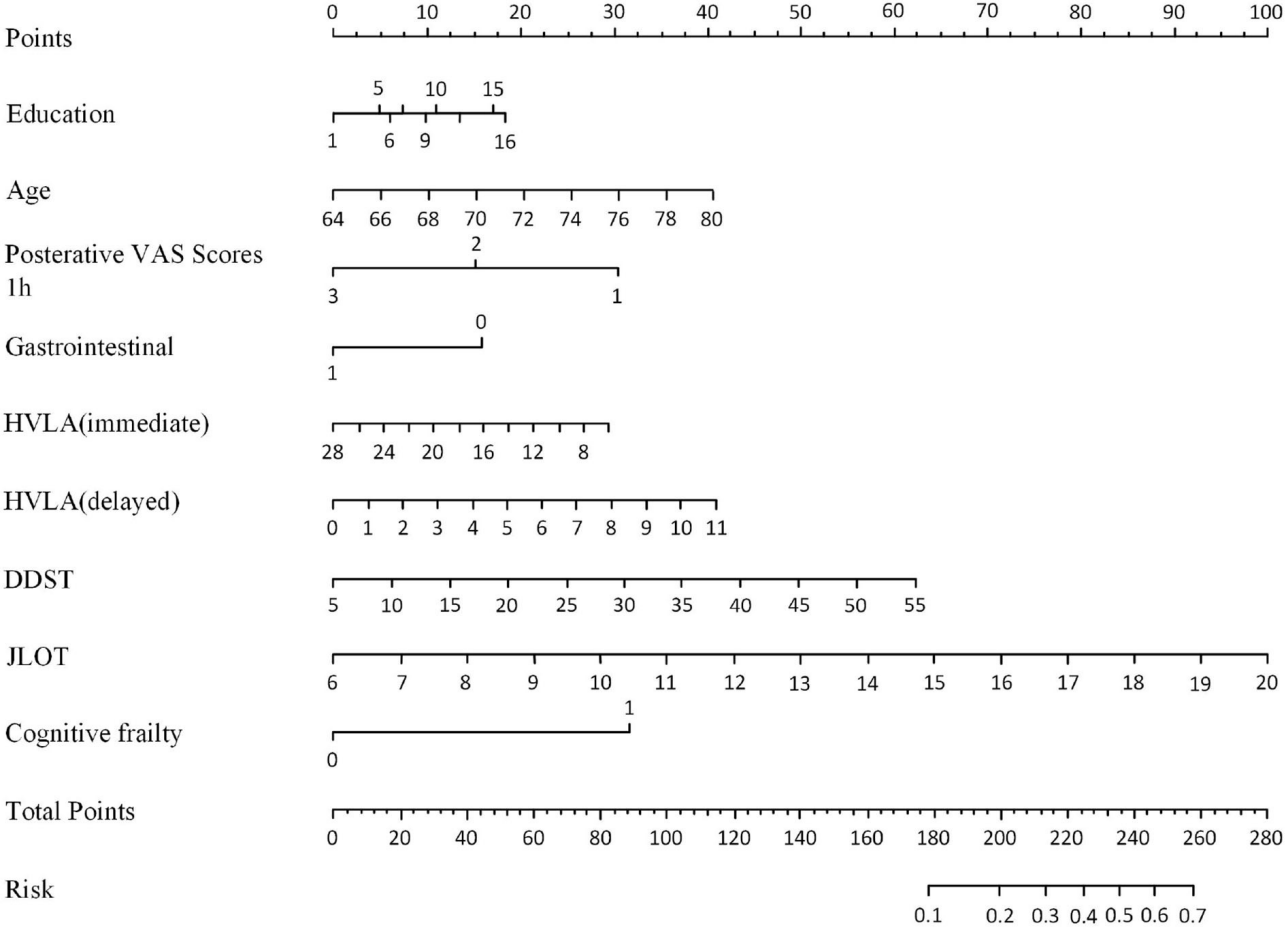
Nomogram for DNR risk and its predictive performance in frail elderly patients undergoing elective noncardiac surgery.

**Figure 3:**
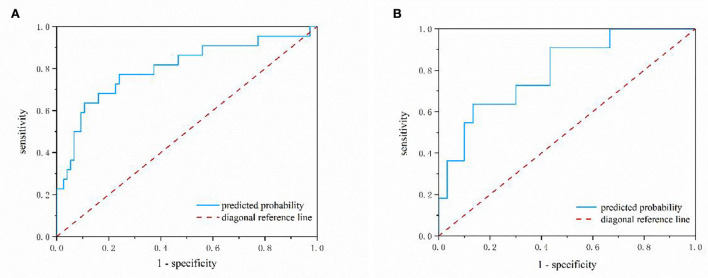
The nomogram model predicting the risk of DNR in frail elderly patients by ROC analysis. **(A)** Modeling group **(B)** validation group.

**Figure 4:**
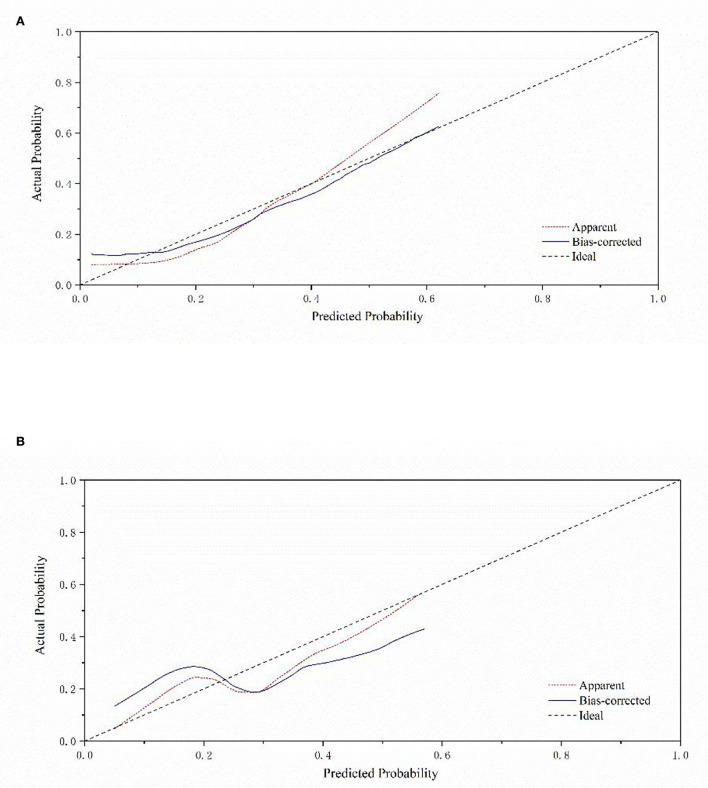
Calibration curve of a nomogram model for predicting the risk of DNR in frail elderly patients. **(A)** Modeling group **(B)** validation group.

Figure 2 depicts a nomogram showing the risk of DNR in frail elderly patients based on multivariate regression analysis results. To explore the predictive value of the predictive probability for DNR, ROC analysis and the calibration curve were performed. As listed in Figure 3, the AUC of the forecast probability in the modeling population (*n* = 97) for DNR was 0.801 (95% CI: 0.683–0.918; Figure 3A), with a sensitivity of 77.3%, a specificity of 76.0%, and a cut-off value of 0.533. In the validation set (*n* = 41), the AUC of the forecast probability was 0.797 (95% CI: 0.646–0.948; Figure 3B), with a sensitivity of 63.6%, a specificity of 86.7%, and a cut-off value of 0.503. The calibration curves of the model population (Figure 4A) and validation population (Figure 4B) showed that the calibration curves of the prediction model were very close to the standard curves. Goodness-of-fit test results indicated that the prediction model does not deviate much from the actual condition: modeling population (*P* = 0.605) and verification population (*P* = 0.484).

## Discussion

As far as we know, this is the first research to investigate the connection between preoperative cognitive frailty and DNR in geriatric patients undergoing elective noncardiac surgery. In this study, contrary to our hypothesis, we did not find an association between the incidence of DNR and cognitive frailty. As noted in previous work, some studies showed no significant difference in the incidence of DNR between MCI and non-MCI patients (Bekker et al., 2010; Trubnikova et al., 2014; Maleva et al., 2020), which indicated that MCI might not be a predictor for DNR. Although some studies have shown that frailty is an essential factor in cognitive dysfunction (Siejka et al., 2022), they are mostly based on community-dwelling elderly samples, and the majority of studies have mainly focused on exploring the relationship between frailty and long-term changes in cognitive function. In our study, we followed up only for a short time for the occurrence of DNR and did not find any association between cognitive frailty and DNR.

DNR is a common postoperative complication following major surgery with unclear specific etiopathogenesis in aging patients (Chan et al., 2013; Eckenhoff et al., 2020). Although methodological issues have plagued the identification of risk factors, numerous prospective, retrospective, and case-control studies have strived to identify risk factors for developing DNR. To explore the risk factors for DNR in frail elderly patients undergoing elective noncardiac surgery, we compared the preoperative data of the DNR group with the non-DNR group (Table 2) and constructed a prediction model for the occurrence of DNR. We calculated a DNR incidence rate of 23.9% (33/138, Table 1), which was within the range of 17–43% reported by Evered et al. (2011). In addition, our findings indicated that the scores of JLOT before surgery were independent risk factors for DNR in frail elderly individuals. The JLOT is one of the most commonly used assessments for evaluating visuospatial perception (Qualls et al., 2000). Generally, JLOT inspects the ability to estimate angular relationships between line segments, a test that requires little or no linguistic mediation and is free of cultural content (Collaer and Nelson, 2002). This study suggested that the assessment of JLOT might improve the accuracy of identification of frail elderly patients at the highest risk of DNR. Thus, our finding supports the possibility of JLOT's role in predicting incident DNR. We highly recommend that the JLOT scores be included in the definition of DNR by a battery of neuropsychological tests.

Paredes and colleagues analyzed 7 of 24 studies and claimed that increasing age was the most common risk factor for DNR (Paredes et al., 2016). However, in this study, we did not find age to be a potential risk factor for DNR, the reason for which we speculated that the incidence of DNR would increase with increasing age (Olotu, 2020). However, the population age we collected mostly ranged from 65 to 75 years old. The age distribution was relatively concentrated, which weakened the relationship of age with DNR. Studies have revealed that a low education level is also a risk factor for DNR (Moller et al., 1998; Lloyd et al., 2012; Brown and Deiner, 2016). However, the similar mean level of education between groups in this study also weakened the relationship between education and DNR.

Moreover, to our knowledge, this is the first research to propose a nomogram model to predict the risk of DNR in frail elderly patients undergoing elective noncardiac surgery. The nomogram model is established based on nine effective indicators, which can conveniently calculate the probability of DNR risks in frail elderly patients. The AUC value of the nomogram model in the training set was 0.794, and in the validation set, the AUC value of the nomogram model was 0.797. The geriatric calibration curve showed that the model-predicted probabilities closely matched the actual probabilities on the training and validation sets. These results suggested that the model had sufficient discriminative ability and high accuracy for the prediction of DNR in frail elderly individuals undergoing elective noncardiac surgery.

Above all, the JLOT test is an independent risk factor for DNR. We suggest that among so many neuropsychological tests, preoperative JLOT evaluation may be the most helpful as a promising predictor for DNR occurrence. We believe that the prediction model established may have certain guiding significance for clinical work to predict the occurrence of DNR in frail elderly patients. To our knowledge, DNR, a common postoperative complication, has a higher incidence rate in elderly patients, resulting in more extended hospital stays, higher costs, more significant social burden, and even higher mortality. Therefore, identifying patients with DNR has important clinical significance for anesthesiologists. For frail elderly patients with an increased risk of DNR assessed by predictive nomogram, timely intervention, frequent dynamic cognitive assessment, more comprehensive postoperative care, and long-term cognitive follow-up are recommended to reduce the risk, which also requires the joint participation of multiple disciplines.

Additionally, we compared the baseline data between the cognitive frailty and non-cognitive frailty groups (Supplementary Table S1), and we found that the two potential risk factors—years of education and hypertension—were significantly different between the two groups. Patients with fewer education years were significantly correlated with cognitive frailty. Wongtrakulruang et al. (2020) also linked low education levels (primary school or less) to a greater incidence of physical frailty and MCI (Wongtrakulruang et al., 2020), as the I.A.N.A–I.A.G.G definition stipulates that cognitive frailty is characterized by a reduction in cognitive reserve, which is largely influenced by education level. Education level has been the only measure of cognitive frailty by far (Facal et al., 2019), which parallels our findings. We also found that patients with hypertension were significantly associated with cognitive frailty. It has been proven that frail elderly adults are accompanied by hypertension. Hypertension is connected with an increased incidence of frailty and accelerated functional decline (Woods et al., 2005). Meanwhile, Walker et al. reported that chronic hypertension had a relationship with an increased risk of cognitive decline (Power et al., 2013; Walker et al., 2017), which is consistent with our findings.

### Limitation

There are some limitations to our research. The small sample size is a major limitation of the current study. The study was conducted in a single center, and difficulties in patient recruitment have resulted in relatively modest sample sizes, which may lead to potential type II errors. Another limitation was that we could not perform lengthier postoperative follow-ups to assist us in establishing the patient's long-term prognosis, as we only had 7 days of follow-up.

## Conclusion

The present study showed that cognitive frailty is not an independent risk factor for predicting delayed neurocognitive recovery after noncardiac surgery in frail elderly individuals. Furthermore, our study demonstrated that the neuropsychological test JLOT is an independent risk factor for early DNR in frail elderly patients. A predictive model for early postoperative DNR in the frail elderly was constructed. The prediction model has a good degree of discrimination and calibration and can individually predict the risk probability of early postoperative DNR in frail elderly patients, which is helpful for medical staff to evaluate quickly and intuitively.

## Data availability statement

The original contributions presented in the study are included in the article/Supplementary material, further inquiries can be directed to the corresponding author.

## Ethics statement

The studies involving human participants were reviewed and approved by the Tongji University's Institutional Review Board (IRB2021-LCYJ-014). The patients/participants provided their written informed consent to participate in this study.

## Author contributions

JZ drafted the manuscript. JiaL conceived and designed the study. JZ, DB, JY, XD, JieL, and FW helped to collect data. JZ and JiaL analyzed the data. DB refined the language. XZ was responsible for quality control. All the authors contributed to the interpretation of the data and the revision of the manuscript and approved the submission of the revision.

## Funding

This study was supported by research grants from the National Natural Science Foundation of China (Nos. 82171194 and 81974155 to JiaL), Clinical Research Plan of SHDC (No. SHDC2020CR1005A to Changhong Miao), Shanghai Science and Technology Commission Biomedical Science Project (No. 22S31902600 to JiaL), and Shanghai Tongji Hospital Youth Project (No. ITJ(QN)2008 to JiaL).

## Conflict of interest

The authors declare that the research was conducted in the absence of any commercial or financial relationships that could be construed as a potential conflict of interest.

## Publisher's note

All claims expressed in this article are solely those of the authors and do not necessarily represent those of their affiliated organizations, or those of the publisher, the editors and the reviewers. Any product that may be evaluated in this article, or claim that may be made by its manufacturer, is not guaranteed or endorsed by the publisher.

## References

[B1] Abellan Van KanG.RollandY.BergmanH.MorleyJ. E.KritchevskyS. B.VellasB. (2008a). The I.A.N.A task force on frailty assessment of older people in clinical practice. J. Nut.r Health Aging. 12, 29–37. 10.1007/BF0298216118165842

[B2] Abellan Van Kan G. Rolland Y. M. Morley J. E. Vellas B. (2008b). Frailty: toward a clinical definition. J. Am. Med. Dir. Assoc. 9, 71–72. 10.1016/j.jamda.2007.11.005 18261696

[B3] Aguayo G. A. Vaillant M. T. Donneau A. F. Schritz A. Stranges S. Malisoux L.. (2018). Comparative analysis of the association between 35 frailty scores and cardiovascular events, cancer, and total mortality in an elderly general population in England: an observational study. PLoS Med. 15, e1002543. 10.1371/journal.pmed.1002543 29584726 PMC5870943

[B4] Androsova G. Krause R. Winterer G. Schneider R. (2015). Biomarkers of postoperative delirium and cognitive dysfunction. Front. Aging. Neurosci. 7, 112. 10.3389/fnagi.2015.00112 26106326 PMC4460425

[B5] Aprahamian I. Cezar N. O. C. Izbicki R. Lin S. M. Paulo D. L. V. Fattori A.. (2017). Screening for frailty with the FRAIL Scale: a comparison with the phenotype criteria. J. Am. Med. Dir. Assoc. 18, 592–596. 10.1016/j.jamda.2017.01.009 28279607

[B6] Bekker A. Lee C. Santi D. E Pirraglia S. Zaslavsky E. Farber A.. (2010). Does mild cognitive impairment increase the risk of developing postoperative cognitive dysfunction? Am. J. Surg. 199, 782–788. 10.1016/j.amjsurg.2009.07.042 20609722 PMC3148659

[B7] Boone M. D. Sites B. Von Recklinghausen F. M. Mueller A. Taenzer A. H. Shaefi S.. (2020). Economic burden of postoperative neurocognitive disorders among US medicare patients. JAMA Netw. Open 3, e208931. 10.1001/jamanetworkopen.2020.8931 32735336 PMC7395237

[B8] Brown C. T. Deiner S. (2016). Perioperative cognitive protection. Br. J. Anaesth. 117, iii52–iii61. 10.1093/bja/aew361 27940456 PMC6857583

[B9] Bu Z. Huang A. Xue M. Li Q. Bai Y. Xu G.. (2021). Cognitive frailty as a predictor of adverse outcomes among older adults: A systematic review and meta-analysis. Brain Behav. 11, e01926. 10.1002/brb3.1926 33159430 PMC7821586

[B10] Chan M. T. Cheng B. C. Lee T. M. Gin T. (2013). BIS-guided anesthesia decreases postoperative delirium and cognitive decline. J. Neurosurg. Anesthesiol. 25, 33–42. 10.1097/ANA.0b013e3182712fba 23027226

[B11] Collaer M. L. Nelson J. D. (2002). Large visuospatial sex difference in line judgment: possible role of attentional factors. Brain. Cogn. 49, 1–12. 10.1006/brcg.2001.1321 12027388

[B12] Collard R. M. Boter H. Schoevers R. A. Oude Voshaar R. C. (2012). Prevalence of frailty in community-dwelling older persons: a systematic review. J. Am. Geriatr. Soc. 60, 1487–1492. 10.1111/j.1532-5415.2012.04054.x 22881367

[B13] Culley D. J. Flaherty D. Fahey M. C. Rudolph J. L. Javedan H. Huang C. C.. (2017). Poor performance on a preoperative cognitive screening test predicts postoperative complications in older orthopedic surgical patients. Anesthesiology 127, 765–774. 10.1097/ALN.0000000000001859 28891828 PMC5657553

[B14] DartiguesJ. F.AmievaH. (2014). Cognitive frailty: rational and definition from an (I.a.N.a./i.a.g.g.) international consensus group. J. Nutr. Health Aging. 18, 95. 10.1007/s12603-013-0437-524402397

[B15] Du X. Gao Y. Liu S. Zhang J. Basnet D. Yang J.. (2021). Early warning value of ASL-MRI to estimate premorbid variations in patients with early postoperative cognitive dysfunctions. Front. Aging. Neurosci. 13, 670332. 10.3389/fnagi.2021.670332 34483876 PMC8416237

[B16] Eckenhoff R. G. Maze M. Xie Z. Culley D. J. Goodlin S. J. Zuo Z.. (2020). Perioperative neurocognitive disorder: state of the preclinical science. Anesthesiology 132, 55–68. 10.1097/ALN.0000000000002956 31834869 PMC6913778

[B17] Evered L. Scott D. A. Silbert B. Maruff P. (2011). Postoperative cognitive dysfunction is independent of type of surgery and anesthetic. Anesth. Analg. 112, 1179–1185. 10.1213/ANE.0b013e318215217e 21474666

[B18] Facal D. Maseda A. Pereiro A. X. Gandoy-Crego M. Lorenzo-López L. Yanguas J.. (2019). Cognitive frailty: a conceptual systematic review and an operational proposal for future research. Maturitas 121, 48–56. 10.1016/j.maturitas.2018.12.006 30704565

[B19] Freitas S. Simões M. R. Alves L. Santana I. (2012). Montreal Cognitive Assessment: influence of sociodemographic and health variables. Arch. Clin. Neuropsychol. 27, 165–175. 10.1093/arclin/acr116 22277128

[B20] Horton D. K. Hynan L. S. Lacritz L. H. Rossetti H. C. Weiner M. F. Cullum C. M.. (2015). An abbreviated montreal cognitive assessment (MoCA) for dementia screening. Clin. Neuropsychol. 29, 413–425. 10.1080/13854046.2015.1043349 25978540 PMC4501880

[B21] Inouye S. K. Leo-Summers L. Zhang Y. Bogardus S. T. Jr. Leslie D. L. Agostini J. V. (2005). A chart-based method for identification of delirium: validation compared with interviewer ratings using the confusion assessment method. J. Am. Geriatr. Soc. 53, 312–318. 10.1111/j.1532-5415.2005.53120.x 15673358

[B22] KelaiditiE.CesariM.CanevelliM.Van KanG. A.OussetP. J.Gillette-GuyonnetS.. (2013). Cognitive frailty: rational and definition from an (I.A.N.A./I.A.G.G.) international consensus group. J. Nutr. Health Aging 17, 726–734. 10.1007/s12603-013-0367-224154642

[B23] Kim S. Brooks A. K. Groban L. (2015). Preoperative assessment of the older surgical patient: honing in on geriatric syndromes. Clin. Interv. Aging 10, 13–27. 10.2147/CIA.S75285 25565783 PMC4279607

[B24] Kotekar N. Shenkar A. Nagaraj R. (2018). Postoperative cognitive dysfunction - current preventive strategies. Clin. Interv. Aging 13, 2267–2273. 10.2147/CIA.S133896 30519008 PMC6233864

[B25] Li Y. L. Huang H. F. Le Y. (2021). Risk factors and predictive value of perioperative neurocognitive disorders in elderly patients with gastrointestinal tumors. BMC Anesthesiol. 21, 193. 10.1186/s12871-021-01405-7 34281529 PMC8287702

[B26] Lloyd D. G. Ma D. Vizcaychipi M. P. (2012). Cognitive decline after anaesthesia and critical care. Conti. Educ. Anaesth. Crit. Care Pain 12, 105–109. 10.1093/bjaceaccp/mks004

[B27] Maleva O. V. Trubnikova O. A. Syrova I. D. Solodukhin A. V. Golovin A. A. Barbarash O. L.. (2020). [Incidence of postoperative cognitive dysfunction after simultaneous carotid surgery and coronary artery bypass grafting in patients with asymptomatic cerebral atherosclerosis]. Zh Nevrol Psikhiatr Im S S Korsakova 120, 5–12. 10.17116/jnevro20201200325 32307423

[B28] Moller J. T. Cluitmans P. Rasmussen L. S. Houx P. Rasmussen H. Canet J.. (1998). Long-term postoperative cognitive dysfunction in the elderly ISPOCD1 study. ISPOCD investigators. International study of post-operative cognitive dysfunction. Lancet 351, 857–861. 10.1016/S0140-6736(97)07382-0 9525362

[B29] Monk T. G. Weldon B. C. Garvan C. W. Dede D. E. van der Aa M. T. Heilman K. M.. (2008). Predictors of cognitive dysfunction after major noncardiac surgery. Anesthesiology 108, 18–30. 10.1097/01.anes.0000296071.19434.1e 18156878

[B30] Nasreddine Z. S. Phillips N. A. Bédirian V. Charbonneau S. Whitehead V. Collin I.. (2005). The montreal cognitive assessment, MoCA: a brief screening tool for mild cognitive impairment. J. Am. Geriatr. Soc. 53, 695–699. 10.1111/j.1532-5415.2005.53221.x 15817019

[B31] Needham M. J. Webb C. E. Bryden D. C. (2017). Postoperative cognitive dysfunction and dementia: what we need to know and do. Br. J. Anaesth. 119, i115–i125. 10.1093/bja/aex354 29161395

[B32] O'Caoimh R. Sezgin D. O'Donovan M. R. Molloy D. W. Clegg A. Rockwood K.. (2021). Prevalence of frailty in 62 countries across the world: a systematic review and meta-analysis of population-level studies. Age Ageing 50, 96–104. 10.1093/ageing/afaa219 33068107

[B33] Olotu C. (2020). Postoperative neurocognitive disorders. Curr. Opin. Anaesthesiol. 33, 101–108. 10.1097/ACO.0000000000000812 31764008

[B34] Paredes S. Cortínez L. Contreras V. Silbert B. (2016). Post-operative cognitive dysfunction at 3 months in adults after noncardiac surgery: a qualitative systematic review. Acta. Anaesthesiol. Scand. 60, 1043–1058. 10.1111/aas.12724 27027720

[B35] Paul P. Pennell M. L. Lemeshow S. (2013). Standardizing the power of the Hosmer-Lemeshow goodness of fit test in large data sets. Stat. Med. 32, 67–80. 10.1002/sim.5525 22833304

[B36] Pinto T. C. C. Machado L. Bulgacov T. M. Rodrigues-Júnior A. L. Costa M. L. G. Ximenes R. C. C.. (2019). Is the montreal cognitive assessment (moca) screening superior to the mini-mental state examination (MMSE) in the detection of mild cognitive impairment (MCI) and Alzheimer's Disease (AD) in the elderly? Int. Psychogeriatr. 31, 491–504. 10.1017/S1041610218001370 30426911

[B37] Power M. C. Tchetgen E. J. Sparrow D. Schwartz J. Weisskopf M. G. (2013). Blood pressure and cognition: factors that may account for their inconsistent association. Epidemiology 24, 886–893. 10.1097/EDE.0b013e3182a7121c 24030502 PMC3818218

[B38] Prins N. D. Scheltens P. (2015). White matter hyperintensities, cognitive impairment and dementia: an update. Nat. Rev. Neurol. 11, 157–165. 10.1038/nrneurol.2015.10 25686760

[B39] Qualls C. E. Bliwise N. G. Stringer A. Y. (2000). Short forms of the benton judgment of line orientation test: development and psychometric properties. Arch. Clin. Neuropsychol. 15, 159–163. 10.1093/arclin/15.2.159 14590559

[B40] Ritt M. Ritt J. I. Sieber C. C. Gaßmann K. G. (2017). Comparing the predictive accuracy of frailty, comorbidity, and disability for mortality: a 1-year follow-up in patients hospitalized in geriatric wards. Clin. Interv. Aging 12, 293–304. 10.2147/CIA.S124342 28223787 PMC5308479

[B41] Siejka T. P. Srikanth V. K. Hubbard R. E. Moran C. Beare R. Wood A. G.. (2022). Frailty is associated with cognitive decline independent of cerebral small vessel disease and brain atrophy. J. Gerontol. A. Biol. Sci. Med. Sci. 9, 1819–1826. 10.1093/gerona/glac078 35363862 PMC9434440

[B42] Sugimoto T. Ono R. Kimura A. Saji N. Niida S. Toba K.. (2019). Cross-sectional association between cognitive frailty and white matter hyperintensity among memory clinic patients. J. Alzheimers. Dis. 72, 605–612. 10.3233/JAD-190622 31594230

[B43] Sugimoto T. Sakurai T. Ono R. Kimura A. Saji N. Niida S.. (2018). Epidemiological and clinical significance of cognitive frailty: a mini review. Ageing Res. Rev. 44, 1–7. 10.1016/j.arr.2018.03.002 29544875

[B44] Trubnikova O. A. Mamontova A. S. Syrova I. D. Maleva O. V. Barbarash O. L. (2014). Does preoperative mild cognitive impairment predict postoperative cognitive dysfunction after on-pump coronary bypass surgery? J. Alzheimers Dis. 42, S45–51. 10.3233/JAD-132540 24898639

[B45] Uslu A. Canbolat O. (2021). Relationship between frailty and fatigue in older cancer patients. Semin. Oncol. Nurs. 37, 151179. 10.1016/j.soncn.2021.151179 34275706

[B46] Walker K. A. Power M. C. Gottesman R. F. (2017). Defining the relationship between hypertension, cognitive decline, and dementia: a review. Curr. Hypertens. Rep. 19, 24. 10.1007/s11906-017-0724-3 28299725 PMC6164165

[B47] Wongtrakulruang P. Muangpaisan W. Panpradup B. Tawatwattananun A. Siribamrungwong M. Tomongkon S.. (2020). The prevalence of cognitive frailty and pre-frailty among older people in Bangkok metropolitan area: a multicenter study of hospital-based outpatient clinics. J. Frailty Sarcopenia Falls 5, 62–71. 10.22540/JFSF-05-062 32885103 PMC7461353

[B48] Woods N. F. LaCroix A. Z. Gray S. L. Aragaki A. Cochrane B. B. Brunner R. L.. (2005). Frailty: emergence and consequences in women aged 65 and older in the women's health initiative observational study. J. Am. Geriatr. Soc. 53, 1321–1330. 10.1111/j.1532-5415.2005.53405.x 16078957

[B49] Zaslavsky O. Cochrane B. B. Thompson H. J. Woods N. F. Herting J. R. LaCroix A. (2013). Frailty: a review of the first decade of research. Biol. Res. Nurs. 15, 422–432. 10.1177/1099800412462866 23086382

