# Supplementary material for: Does cognitive frailty predict delayed neurocognitive recovery after noncardiac surgery in frail elderly individuals? Probably not
Source: Front Aging Neurosci. 2022 Nov 15;14:995781. doi: 10.3389/fnagi.2022.995781 (PMC9705765; doi:10.3389/fnagi.2022.995781)
Supplement: Supplementary file 1 [file Table_1.DOCX]

Supplement table 1 Demographic and clinical data related to cognitive frailty in elderly patients

| Item | CF(n=79) | NCF(n=59) | P-value |
| --- | --- | --- | --- |
| Male (n, %) | 49(62.0%) | 39(66.1%) | 0.622 |
| Age(y), median [Q25, Q75] | 69[65,73] | 67[70 72] | 0.552 |
| Education(y),  median [Q25, Q75] | 9[6 9] | 10[9 12] | <0.0001* |
| BMI (kg/m2), mean ± SD | 24.3±3.7 | 24.6±3.1 | 0.628 |
| Surgery time(min),  median [Q25, Q75] | 165[135 215] | 145[175 210] | 0.292 |
| Anesthesia time(min),  median [Q25, Q75] | 210[180 270] | 165[200 255] | 0.305 |
| BIS, mean ± SD | 50.4±5.6 | 50.5±5.7 | 0.848 |
| DNR (n, %) | 19(24,1%) | 14(23.7%) | 0.965 |
| Postoperative VAS scores,  median [Q25, Q75] |  |  |  |
| 1h | 2[2 3] | 2[2 3] | 0.396 |
| 12h | 1[1 2] | 1[1 2] | 0.462 |
| 24h | 0[0 1] | 0[0 1] | 0.102 |
| 48h | 0[0 0] | 0[0 0] | 0.140 |
| Postoperative Resmay scores,  median [Q25, Q75] |  |  |  |
| 1h | 1[1 2] | 1[1 2] | 0.136 |
| 12h | 3[3 3] | 3[3 3] | 0.591 |
| Medical history (n, %) |  |  |  |
| Hypertension | 50(63.3%) | 23(40%) | 0.005* |
| Diabetes | 13(16.5%) | 13(22.0%) | 0.407 |
| Cardiovascular Disease | 17(21.5%) | 10(17.0%) | 0.503 |
| Chronic Pulmonary Diseases | 3(4.0%) | 5(8.0%) | 0.287 |
| Surgery category (n, %) |  |  |  |
| Spine | 36(45.6%) | 34(57.6%) | 0.161 |
| Urinary | 21(26.6%) | 14(23.7%) | 0.586 |
| Gastrointestinal | 22(27.8%) | 11(18.9%) | 0.210 |

BMI: *body mass index*; CF: *cognitive frailty*; MMSE: *Mini-mental state examination*; MoCA: *Montreal cognitive assessment*; VAS: *visual analogue scale*
